# Gene-microRNA network module analysis for ovarian cancer

**DOI:** 10.1186/s12918-016-0357-1

**Published:** 2016-12-23

**Authors:** Shuqin Zhang, Michael K. Ng

**Affiliations:** 1 Center for Computational Systems Biology, School of Mathematical Sciences, Fudan University, No.220 Handan Road, Shanghai, 200433 China; 2 Department of Mathematics, Hongkong Baptist University, Kowloon Tong, Hongkong, Hongkong

**Keywords:** Gene-miRNA network, Module identification, Data integration

## Abstract

**Background:**

MicroRNAs (miRNAs) are involved in many biological processes by regulating post-transcriptional gene expression. The alterations of the regulatory pathways can cause different diseases including cancer. Although many works have been done to study the gene-miRNA regulatory network, the intertwined relationship is far from being fully understood. The objective of this study is to integrate both gene expression data and miRNA data so as to explore the complex relationships among them.

**Methods:**

By integrating the networks consisting of gene coexpression, miRNA coexpression, gene-miRNA coexpression, and the known gene-miRNA interactions, we aim to find the most connected network modules so as to study their functions and properties. In this paper, we proposed an optimization model for identification of the modules in the integrated networks. This model tries to find both the modules in the gene-gene and miRNA-miRNA coexpression networks and the densely connected gene-miRNA subneworks. An approximation computational method was developed to solve the optimization problem.

**Results:**

We applied the method to 556 human ovarian cancer samples with both gene expression data and miRNA expression data. The identified modules are significantly enriched by miRNA clusters, GO-BPs, and KEGG pathways. We compared our method with some existing methods and showed the better performance of our method. We also showed that the miRNAs and genes in our identified modules are associated with cancers, especially ovarian cancer.

**Conclusions:**

This study provides strong support that the subnetworks consisting of genes and miRNAs with close interactions contribute the cancers. The proposed computational method can be applied to other studies that are related to different types of networks.

**Electronic supplementary material:**

The online version of this article (doi:10.1186/s12918-016-0357-1) contains supplementary material, which is available to authorized users.

## Background

MicroRNAs (miRNAs) are small (’22 nucleotides) non-coding RNAs that have emerged as key gene regulators in diverse plant and animal genomes. Typically, miRNAs regulate the genes by base pairing with the complementary sequences of the corresponding mRNAs, either inhibiting translation or degrading the mRNAs [1, 2]. MiRNAs are involved in many biological processes, such as development, differentiation, apoptosis and proliferation [3–6]. Each miRNA is potentially able to regulate around 100 or more mRNA targets and over 30% of all human genes are supposed to be regulated by miRNAs [3, 6, 7]. The alterations in the regulatory pathways can cause different diseases, including cancer, heart disease, cardiovascular disease, and matabolc disorders [8–13]. The disruption of the miRNA functions will contribute to these diseases. Therefore, identification and validation of miRNA targets is essential, which may lead to new therapeutic methods [6, 14–16].

MiRNA targets prediction has attracted much attention in recent years. Although many experimental tools for miRNA target validation are available [17–22], the lack of high-throughput and low-cost methods makes the development of computational techniques necessary. The computational methods are mainly based on expression data of both miRNAs and mRNAs and the sequence-based putative interactions between them. Roughly, these methods can be divided into three groups. The first group includes methods that compute the pairwise correlations or mutual information between miRNAs and mRNAs [23–30]. The second group is mainly related to the linear regression methods [31–35]. And the third group is mainly based on Bayesian methods [36–39]. A review of these methods can be found in [6].

Although huge advances have been made for miRNA target prediction, a lot of works are still left to do. Most of the current methods mainly considered the down-regulatory effects from miRNAs. However, as shown in [6], by considering the enhancement effects from miRNAs, more interactions are identified and biologically sound. Also, as shown in [40], the base pairing between an miRNA and its target not only results in repressed target expression, but also have an impact on the levels of the miRNA. And as one miRNA may regulate many mRNAs, and be regulated by several miRNAs, the intertwined relationship between miRNAs and mRNAs becomes very complex. Therefore, the systems tools such as networks should be more appropriate for studying the relationships between miRNAs and mRNAs.

Recently, a few papers have been published to study the complex interactions between genes and miRNAs from the system point of view by using networks [41–43]. One essential property of many different types of networks is the module structure, which describes the densely connected subnetworks. The members in the same module may function as a whole in the system. By identifying the modules, we can do gene prediction, gene function annotation and so on. In the networks composed of both genes and miRNAs, a good module should include both genes and miRNAs with connections. To better identify the gene-miRNA modules, Zhang et al. developed a framework of SNMNMF, which is based on non-negative matrix factorization and utilized a variety of data, including gene-gene interaction (GGI) and transcription factor binding sites (TFBS) [41]. However, SNMNMF tends to pay more attention to the gene modules while overlook the connections between miRNAs. Also, its computational speed may limit the practical use of the method. Then, using similar datasets, Le et al. described a regression-based method, PIMiM36 (Protein Interaction based MiRNA Modules) [42], but using a non-convex algorithm. This method may result in unstable outcomes because of its random initialization. Then, Li et al. developed a two-stage overlap clustering method, Mirsynergy [43]. This method improves the efficiency substantially, and importantly, facilitates the setting of predefined parameters. However, this method does not consider the relations between miRNAs, and thus finds the modules with a large number of genes/miRNAs, which are shown in their enrichment analysis.

In this paper, we establish networks to explore gene-miRNA relationships. We first integrate gene expression and miRNA expression data by measuring the distance between genes, miRNAs and gene-miRNAs with Pearson correlation coefficient, thus transferring all the relations into edges in networks. Based on these networks, we propose our module identification method, and study the gene-miRNA interactions. We also include the known gene-miRNA interactions in our study. In the second section, we will present our method for identifying the modules in the integrated networks. Then we apply the method to the ovarian cancer data to show its performance. We also compared our method with Mirsynergy. Finally, we give our conclusion.

## Methods

Assume we have gene expression data of *N*_*g*_ genes, and miRNA expression data of *N*_*m*_ miRNAs for *N* samples. The first step is to build the coexpression networks. We use Pearson correlation coefficient to measure the coexpressions. After computing the correlations of genes, miRNAs, and gene-miRNAs, we construct the adjacency matrix by hard thresholding. If the absolute value of the Pearson correlation coefficient between genes(miRNAs, gene-miRNAs) is greater than some given value, we assign an edge between them; otherwise, there is no edge. For gene coexpression network and miRNA coexpression network, we try different thresholds and compute the linear regression coefficient between the log10 transformed degree frequency of degree *d* (log10*f*(*d*)) and *d* (log10*d*) to make the network has approximately scale free property as described in [44]. The threshold for constructing the gene-miRNA network depends on the AUCs for the known gene-miRNAs interactions being clustered in the same module. Given a fixed threshold, we use our method proposed in the following to get the score of one gene and one miRNA in the same module. We rearrange the gene-miRNA interactions in the descending order according to their scores and compute the AUC as $\text {AUC}=\frac {\sum _{i=1}^{q} R_{i}-q(q+1)/2}{pq},$ where {*R*_*i*_} is the rank of the *i*_*th*_ known interacting gene-miRNA pair ranking from the smallest, *p* is the number of known non-interacting gene-miRNA pairs, and *q* is the number of known interacting gene-miRNA pairs. We choose the threshold that achieves the highest AUC.

We consider the constructed gene coexpression network *G*_*g*_ and the miRNA coexpression network *G*_*m*_. The adjacency matrix for network *G*_*g*_ is *A*_*g*_, where *A*_*g*_(*i,j*)=1 represents there is an edge between gene *i* and gene *j*. Similarly, we define the adjacency matrix *A*_*m*_ for the miRNA coexpression network. We use *D*_*g*_, *D*_*m*_ to denote the diagonal matrix with the diagonal entries being the degree of the corresponding gene/miRNA, where the degree of gene *i* is defined as $d_{g}(i)=\sum _{j=1}^{N_{g}}A_{g}(i,j)$. The interaction matrix between the genes and miRNAs is denoted as $C_{N_{g}\times N_{m}}$, where *C*(*i,j*)=1 represents there is a connection between the corresponding gene and miRNA. In our study, *C* includes two parts: the gene-miRNA coexpression network, and the known gene-miRNA interaction network. The integrated network is composed of the above four networks. Here, we assume the integrated network is connected. If the network is unconnected, we may divide it into connected parts with some classical methods like spectral clustering. We define the modules of the integrated network to be the densely connected subnetworks consisting of both genes and miRNAs. Assume there are *K* modules in the network. We let *S*_*g*_ be the assignment of the *N*_*g*_ genes into *K* modules for the network *G*_*g*_,

$$\begin{array}{@{}rcl@{}} {S}_{g}(i,k)=\left\{ \begin{array}{ll} 1, &\text{if\,\,vertex\,\,} i\in V_{k}, \\ 0, & \text{otherwise}, \end{array} \right. \end{array} $$

where *i*=1,2,⋯,*N*_*g*_;*k*=1,2,⋯,*K,V*_*k*_ denotes the *k*-th module. Similarly, we define the assignment of the *N*_*m*_ miRNAs into *K* modules for the network *G*_*m*_ as *S*_*m*_.

For each of the two coexpression networks, we may use a module identification method [45] to cluster the genes/miRNAs separately. This method has shown to outperform most updated methods including spectral clustering in module identification. Taking our considered network *G*_*g*_ as an example, we define

1 $$\begin{array}{@{}rcl@{}} \Psi_{g}(S_{g})=\sum_{k=1}^{K}\frac{{S_{g}({.,k})}^{T}(2A_{g}-D_{g}){S}_{g}({.,k})}{{S_{g}({.,k})}^{T}{S}_{g}({.,k})}, \end{array} $$

where *S*_*g*_(.,*k*) denotes the *k*-th column of matrix *S*_*g*_ for the network *G*_*g*_. Then the optimization problem for identifying the modules is formulated as:

2 $$\begin{array}{@{}rcl@{}} \max \,\,&& \,\,\Psi_{g}(S_{g})\\ s.t.\,\, &&\,\, {S}_{g}(i,k)\in \{0,1\},\,\, i=1,2,\cdots,N_{g}, k=1,2,\cdots,K,\\ &&\,\,\sum_{k=1}^{K}{S}_{g}({.,k})=\mathbf{1}, \end{array} $$

where **1** is a vector with all the entries being 1. By letting $\tilde {S}_{g}({.,k})=\frac {{S}_{g}({.,k})}{\|{S}_{g}({.,k})\|_{2}}$, the problem is relaxed to:

$$\max \,\, {\tilde\Psi_{g}}({\tilde S_{g}})=\text{Tr }\left(\tilde {S_{g}}^{T}(2A_{g}-D_{g})\tilde S_{g}\right)\quad s.t. \,\, \tilde {S_{g}}^{T}\tilde {S_{g}}=I_{K}. $$

Let *L*_*g*_=2*A*_*g*_−*D*_*g*_, we can use the standard procedure of spectral clustering to get the module label for the network.

Similarly, we can define the optimization problem for clustering the miRNAs in the miRNA coexpression network. We use *Ψ*_*m*_(*S*_*m*_) to denote the objective function when doing module identification for miRNAs, and define $\tilde S_{m}(.,k)=\frac {S_{m}(.,k)}{\|S_{m}(.,k)\|_{2}}$. To find the modules in the integrated network, besides considering the connections in both gene coexpression and miRNA coexpression networks, we expect that the genes and miRNAs with dense connections are clustered into one module. That is, we want to maximize *S*_*g*_^*T*^(.,*k*)*CS*_*m*_(.,*k*). To balance the size of genes and miRNAs in different modules, we divide the term ${{S_{g}^{T}}}({.,k})C{S_{m}}({.,k})$ by ∥*S*_*g*_(.,*k*)∥_2_∥*S*_*m*_(.,*k*)∥_2_. By putting all these terms together, our objective becomes:

$$\begin{aligned} {\Psi}(S_{g},S_{m})=&\, {\Psi_{g}}(S_{g})+{\Psi_{m}}(S_{m})\\&+\lambda\sum_{k=1}^{K}\frac{{{S_{g}^{T}}}({.,k})C{S_{m}}({.,k})}{\|{S_{g}}^{T}({.,k})\|_{2}\|{S_{m}}({.,k})\|_{2}}, \end{aligned} $$

where *λ* controls the contributions of the connections within each coexpression network and those between the two networks. The optimization problem is formulated as:

$$\begin{array}{@{}rcl@{}} \max &&\,\, {\Psi}(S_{g},S_{m})\\ s.t. &&\,\, S_{g}({i,k})\in \{0,1\},i=1,2,\cdots,N_{g}, k=1,2,\cdots,K,\\ &&\,\, S_{m}({j,k})\in \{0,1\},j=1,2,\cdots,N_{m}, k=1,2,\cdots,K,\\ &&\,\,\sum_{k=1}^{K}{S}_{g}({\cdot,k})=\mathbf{1},\sum_{k=1}^{K}{S}_{m}({\cdot,k})=\mathbf{1}. \end{array} $$

We define $L_{g}=2A_{g}-D_{g}, L_{m}=2A_{m}-D_{m}, L_{w}=diag(L_{g}, L_{m}), L_{b}=\left (\begin {array}{cccc} {\bf {0}}&C\\ C^{T}&\mathbf {0} \end {array} \right)$, $\tilde S=\left (\begin {array}{c} \tilde S_{g}\\ \tilde S_{m}\\ \end {array} \right)$, and *L*=*L*_*w*_+*λ* *L*_*b*_.

With the same technique as in (2), the above optimization problem can be relaxed to:

$$\max \,\, {\tilde\Psi}(\tilde S)=\text{Tr}(\tilde S^{T}L\tilde S),\,\,\,\, s.t. \,\, \tilde S^{T}\tilde S=2I_{K}. $$

In this formulation, the constant coefficient 2 can be put into *L*, such that each column of $\tilde S$ has the norm 1. We take $\tilde S$ as a data set composed of *N*_*g*_+*N*_*m*_ nodes and do *k*-means clustering to get the assignment label for each node.

The algorithm is summarized in the following.



With this algorithm, we can identify the modules in the integrated network. Here, *K* is prespecified as the number of modules in the integrated network. Since any node is assigned to a module, in practice, the nodes in some module may not connect densely. Additionally, some module may consist of genes or miRNAs only. Therefore, after implementing our algorithm, we need to check the structure of the clusters to make sure the identified modules are all densely connected subnetworks with both genes and miRNAs.

## Results

### Data sets

We downloaded the level 2 gene expression and miRNA expression data for ovarian cancer from The Cancer Genome Atlas (TCGA). The gene expression data is generated with UNC AgilentG4502A_07_03, and the miRNA expresssion data is generated with UNC miRNA_8x15kv2. For the gene expression data, we averaged the expression data for different probes corresponding to the same gene, and used the average of different samples to represent the missing data for a specific gene. After the preprocessing, we have 22747 genes with annotations for the 562 samples. We did the same process for the miRNA expression data, and finally we have 590 miRNAs for the 595 samples. We chose the data for the common 556 samples. We downloaded the gene-miRNA interaction data from miRTarBase (*http://mirtarbase.mbc.nctu.edu.tw*). There are a total of 39110 gene-miRNA interactions.

### Network construction

**Fig. 1:**
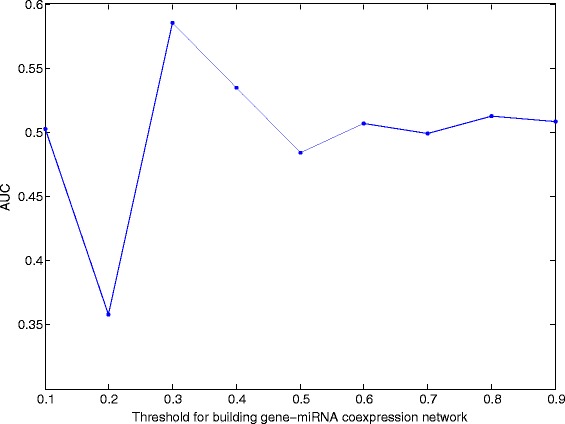
AUCs for different cutoffs when building the gene-miRNA coexpression network

We computed the variance of the expression values for all genes across the considered samples, and selected those genes with large variance. Here, we selected the first 3200 genes with the largest expression variance, which corresponds to the variance greater than 1. We used the method described in the “Methods” section to build the gene coexpression and miRNA coexpression network. We chose a threshold of 0.60 such that the degree of both coexpression networks follows power law distribution, and the main correlations are kept. The average degree of the gene coexpression network and miRNA coexpression network is 13.25 and 8.80, repectively. Here, we set *λ*=1. By choosing the threshold for building gene-miRNA coexpression network, we aim to get a good clustering of genes and miRNAs. We choose the threshold from 0.1 to 0.9 with a stepsize 0.1. For each value, we build the gene-miRNA coexpression network. Then we run our algorithm by setting *C* being this network adjacency matrix. After we get the matrix *T* as shown in our algorithm, we normalize each row of *T* denoted as $\tilde T$ and compute $\tilde T\tilde T^{T}$. It is easy to figure out that the *ij*-th score in $\tilde T\tilde T^{T}$ describes the possibility of the *i*-th subject and the *j*-th subject in the same module. We set the number of modules *K* to be 100 to 200 with a stepsize 10, and compute all the AUCs. Figure 1 shows the average AUCs for different *K*. When the threshold is 0.3, we can cluster the known gene-miRNAs in the same module with the highest AUC of value 0.59. Thus we choose the threshold to be 0.3. The mean of the absolute value of correlation coefficients is 0.07(gene-miRNA), 0.10(gene-gene), and 0.12(miRNA-miRNA). We searched the corresponding interactions between the 3200 genes and the 590 miRNAs in our downloaded interaction data set, and totally there are 2648 interations.

Then the matrix *C* is composed of two types of elements: the known gene-miRNA interactions and the gene-miRNA coexpression network. We note that the best *λ* and threshold can be chosen by changing the values of *λ* and the threshold alternatively, and those achieving the highest AUC are selected.

### Experimental results

We applied our proposed method to the integrated network. For the number of modules, we selected different values starting from 100 to 200. These different values correspond to the modules on different connection levels. We choose those clusters which satisfy: (1) both genes and miRNAs are in the cluster, (2) all of gene-gene, miRNA-miRNA, and gene-miRNA connections appear in the cluster, as our identified modules. With the different choices of number of modules, one gene/miRNA may belong to different modules. We combine those modules that have an overlapping percentage larger than 90%. Finally, we got 46 modules. The full list of identified modules is in Additional file 1.

#### MiRNA module enrichment analysis

We downloaded miRNA cluster data from the miRBase website (*http://www.mirba se.org/*), with the inter-miRNA distance cutoff of 10 kb. This criterion resulted in 153 clusters containing from 2 to 46 miRNAs. In this section, ‘cluster’ means these clusters.

We compared our identified miRNA modules that are included in the gene-miRNA modules with the downloaded clusters. We did enrichment for both clusters and modules to see whether the modules are enriched by the clusters, and whether the clusters are enriched by the modules. We use hypergeometric distribution to do the test, and then use Bonferroni correction to adjust the *p*-values. There are a total of 14 modules enriched by clusters, and 8 clusters enriched by modules with the overlap size between clusters and modules being at least 3. Table 1 shows the information of the enriched modules by different clusters. The “No.” of modules is the corresponding column in Additional file 1. The column “MiRNAs” lists the overlapping miRNAs with the corresponding clusters. The loci of the clusters are also given. One typical example is our identified module 22. All the nodes in the same cluster belong to this module. The total distance for all these miRNAs is around 6000 bp. Taking as another example, 5 of 12 miRNAs in module 35 belong to a cluster with size 6 in Chr13. The total distance of this cluster is about 700 bp.

**Table 1:** MiRNA module enrichment results

No.	*p*-value	MiRNAs	Loci
38	2.01E-18	miR-411, miR-299, miR-758, miR-329-1, miR-543, miR-495,	Chr14 101022066-
		miR-654, miR-376b, miR-376a-1, miR-381, miR-487b, miR-539,	101066801
		miR-487a, miR-382, miR-154, miR-377, miR-409, miR-369,	
		miR-376c,miR-889,miR-410	
45	1.41E-14	miR-379, miR-411, miR-299, miR-758, miR-329-1, miR-543,	Chr14 101022066-
		miR-376c, miR-654, miR-376b, miR-376a-1, miR-381,	-101066801
		miR-487a, miR-382, miR-154, miR-377, miR-409, miR-369,	
		miR-495, miR-487b, miR-539, miR-410	
5	1.29E-15	miR-411, miR-758, miR-329-1, miR-543, miR-495,	Chr14 101022066-
		miR-376b, miR-376a-1, miR-487b„ miR-539, miR-889,	-101066801
		miR-382 miR-154, miR-409, miR-369,	
		miR-654,miR-487a, miR-410	
2	2.19E-02	miR-379, miR-299, miR-376c, miR-376a-1, miR-381, miR-377	Chr14 101022066-
			-101062118
10	1.40E-03	miR-379, miR-299, miR-376c, miR-376a-1, miR-381, miR-377	Chr14 101022066-
			-101062118
38	2.70E-05	miR-493, miR-337, miR-433, miR-127, miR-432, miR-136	Chr14 100869060-
			-100884783
12	1.40E-03	miR-379, miR-299, miR-376c, miR-376a-1, miR-381, miR-377	Chr14 101022066-
			-101062118
21	1.26E-02	miR-379, miR-299, miR-376c, miR-376a-1, miR-381, miR-377	Chr14 101022066-
			-101062118
35	1.17E-06	miR-17, miR-18a, miR-19a, miR-20a, miR-19b-1	Chr13 91350605-
			-91351391
45	5.66E-03	miR-493, miR-337, miR-433, miR-127, miR-136	Chr14 100869000-
			-100885000
18	2.13E-04	miR-424, miR-503, miR-542, miR-450a-1	ChrX 134546614-
			-134540262
1	4.45E-05	miR-200b, miR-200a, miR-429	Chr1 1167104-
			-1169087
10	2.02E-02	miR-337, miR-127, miR-136	Chr14 100869060-
			-100884783
11	1.78E-03	miR-18b, miR-20b, miR-363	ChrX 134170198-
			-134169452
22	2.83E-03	miR-508, miR-507, miR-506	Chr X 147236913-
			-147230843
35	9.44E-04	miR-106b, miR-93, miR-25	Chr 7 100093993-
			-100093643
37	9.44E-04	miR-106b, miR-93, miR-25	Chr 7 100093993-
			-100093643
41	1.40E-02	miR-17, miR-19a, miR-20a	Chr 13 91350605-
			-91351135

#### Gene module enrichment analysis

To evaluate the performance of our proposed method for gene module identification, we did enrichment analysis for Gene Ontology biological process (GO-BP) terms and KEGG pathways with DAVID [46, 47]. By taking the cutoff of the Benjamini *p*-values as 0.05, 15 modules are enriched by GO-BP terms and 7 modules are enriched by KEGG pathways significantly. Table 2 listed the enriched KEGG pathways. All the *p*-values are Benjamin *p*-values from DAVID. The KEGG pathway enrichment results of the modules in Table 1 are also listed. Two typical modules are module 41 and module 18, which have significant enrichment of GO-BP, KEGG pathways and miRNAs clusters. From the KEGG pathway enrichment results, it can be seen that these two modules are quite related to cancers. We put all these enrichment results in Additional file 3.

**Table 2:** Enriched KEGG pathways for the gene modules

No.	Enriched Pathways	*p*-value
17	Cytokine-cytokine receptor interaction	7.40E-05
	NOD-like receptor signaling pathway	1.20E-03
	Chemokine signaling pathway	5.80E-03
	Hematopoietic cell lineage	3.30E-02
	Complement and coagulation cascades	1.80E-01
	Systemic lupus erythematosus	2.70E-01
18	p53 signaling pathway	3.50E-03
	Small cell lung cancer	2.70E-03
	Cell cycle	4.00E-03
	Pathways in cancer	2.10E-02
	Non-small cell lung cancer	8.20E-02
	Glioma	8.00E-02
	Melanoma	7.70E-02
	Pancreatic cancer	6.90E-02
	Chronic myeloid leukemia	6.40E-02
	Prostate cancer	6.80E-02
24	Cytokine-cytokine receptor interaction	4.40E-05
	NOD-like receptor signaling pathway	9.40E-04
	Chemokine signaling pathway	4.30E-03
	Hematopoietic cell lineage	2.70E-02
	Complement and coagulation cascades	1.60E-01
	Systemic lupus erythematosus	2.40E-01
28	Antigen processing and presentation	4.00E-03
	Cytokine-cytokine receptor interaction	1.20E-02
	Natural killer cell mediated cytotoxicity	9.20E-02
	Hematopoietic cell lineage	1.30E-01
	Graft-versus-host disease	1.70E-01
	Chemokine signaling pathway	1.40E-01
	NOD-like receptor signaling pathway	2.70E-01
	Viral myocarditis	3.00E-01
31	Systemic lupus erythematosus	1.30E-02
41	Small cell lung cancer	1.30E-03
	Chronic myeloid leukemia	3.10E-02
	Pathways in cancer	2.40E-02
	Colorectal cancer	1.90E-02
	Cell cycle	3.40E-02
	Thyroid cancer	1.30E-01
	Bladder cancer	1.60E-01
	Endometrial cancer	1.80E-01
	Non-small cell lung cancer	1.60E-01
	Acute myeloid leukemia	1.60E-01
	Glioma	1.60E-01
	p53 signaling pathway	1.50E-01
	Melanoma	1.50E-01
	Pancreatic cancer	1.40E-01
	Prostate cancer	1.60E-01
43	ECM-receptor interaction	1.20E-09
	Focal adhesion	6.70E-06
	Vascular smooth muscle contraction	1.60E-01
21	Focal adhesion	8.80E-01
22	ECM-receptor interaction	8.30E-01
35	Acute myeloid leukemia	8.20E-01
	p53 signaling pathway	6.40E-01
	Chronic myeloid leukemia	5.20E-01
37	Hypertrophic cardiomyopathy (HCM)	7.00E-02
	Gap junction	2.70E-01
	Dilated cardiomyopathy	2.10E-01
	Arrhythmogenic right ventricular cardiomyopathy (ARVC)	4.60E-01
	MAPK signaling pathway	7.80E-01
45	Pathways in cancer	7.30E-01
	Basal cell carcinoma	5.00E-01
	Hedgehog signaling pathway	3.70E-01

#### Gene-miRNA modules are strongly associated with cancers

We checked the 14 modules that are enriched by miRNA clusters, of which 7 modules have enrichment of cancer related pathways. From the KEGG pathway enrichment results, we can directly see that module 18, 41 are associated with different cancers. For module 21, the ‘focal adhesion’ pathway is known to be involved in tumour formation and progression [48]. The pathway ‘ECM-receptor interaction’ enriched by module 22 is also identified to be linked to carcinogenesis in multiple cancers [49]. Since the discovery of the MAPK signalling pathway, which is enriched in module 37, the enormous role of perturbed MAPK signaling in cancer biology has become evident. Specifically, more than 30% of human cancers include mutations in genes encoding proteins in this pathway [50]. Such evidence further shows that the modules enriched by miRNA clusters are likely to be enriched by cancer related pathways.

We checked the cancer related miRNAs from the website: *http://mircancer.ecu.edu*. There are 295 different miRNAs related to cancer, of which 122 are in our identified modules. 57 of the 295 miRNAs are related to ovarian cancer, of which 29 are in our identified modules, which achieves a *p*-value 0.0386. This suggests that the modules we identified are related to ovarian cancer significantly.

**Fig. 2:**
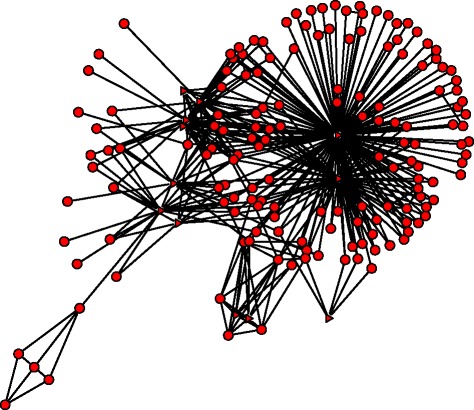
Network structure of module 37. Triangle represents the miRNAs and circle represents the genes

**Fig. 3:**
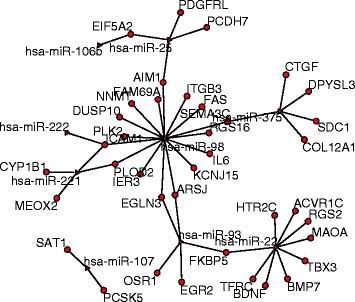
Known interaction network for module 37. Triangle represents the miRNAs and circle represents the genes

For the modules listed in Table 1, all of them have cancer related miRNAs. We listed the number of cancer associated miRNAs in Table 3. 13 of the 14 modules are enriched by cancer associated miRNAs significantly. In module 1 and module 37, all the involved miRNAs are associated with cancers. Figure 2 shows our constructed network for module 37. 156 genes are regulated by the 12 miRNAs. There are a total of 47 known regulations. Figure 3 shows the known regulatory interactions of genes and miRNAs in module 37. From this known network and our module information, we may predict other regulations in this module. In module 1, all the miRNAs are associated with ovarian cancer. The genes in this module take part in the process of transcription, gene expression etc.. The complex gene-miRNA regulatory relations may be related to ovarian cancer. In module 41, 5 miRNAs are associated with ovarian cancer. By checking the GO-BP terms, we found that the most enriched term is ‘sexual reproduction’, which has a *p*-value 7.50E-06, and Benjamini *p*-value 5.40E-03. This module also enriches the GO-term: ‘gamete generation’, ‘male gamete generation’, and ‘spermatogenesis’ significantly. All these show that this module should be very important in ovarian cancer development.

**Table 3:** Cancer associated miRNAs in the modules shown in Table 1

Module No.	1	2	5	10	11	12	18
Total No. of miRNAs	5	13	21	9	6	9	12
No. of cancer miRNAs	5	9	8	6	5	6	10
*p*-value	0	1.80E-04	4.82E-02	3.40E-03	1.77E-03	3.40E-03	4.74E-06
Module No.	21	22	35	37	38	41	45
Total No. of miRNAs	11	11	12	12	29	11	39
No. of cancer miRNAs	7	2	10	12	13	10	15
*p*-value	2.17E-03	7.00E-01	4.74E-06	0	2.29E-03	9.58E-07	6.43E-03

#### Comparison with Mirsynergy

There have been some other methods proposed for studying gene-miRNA modules. SNMNMF is the first paper to address this problem [41], and Mirsynergy works the best till now, to the best of our knowledge. As shown in [43], Mirsynergy works better than SNMNMF, and it runs much faster. Thus here we only compare our method with Mirsynergy. This method operates in two steps: it first detects the miRNA modules based on gene-miRNA relationship, then expands each miRNA module by greedily including (excluding) mRNAs into (from) the miRNA module to maximize the synergy score, which is a function of gene-miRNA and gene–gene interactions. Different from our method, it did not consider the coexpressions of miRNAs. We use the same gene coexpression network, and the same gene-miRNA interaction data including both the known interactions and the gene-miRNA coexpressions. We directly applied the R package Mirsynergy to test the data. Table 4 shows the results.

**Table 4:** Module enrichment performance of Mirsynergy and our method

Method	*N*_module_	${\bar N}_{g}$	${\bar N}_{m}$	*N*_en-module_	*N*_en-cluster_	*N*_GO_	*N*_KEGG_
Mirsynergy	18	88.4	21.9	1	1	4	1
Our method	46	42.6	10.3	14	8	15	7

‘ *N*_module_’ denotes the total number of modules identified. ‘${\bar N}_{g} $’ and ‘${\bar N}_{m} $’ denote the mean number of genes and miRNAs in the modules. ‘ *N*_en-module_’ denotes the number of enriched modules by clusters. ‘ *N*_en-cluster_’ denotes the number of enriched clusters by modules. ‘ *N*_GO_’, ‘ *N*_KEGG_’ denote the number of modules enriched by GO-BP and KEGG pathway

With our proposed method, we identified 46 modules, while with Mirsynergy we identified 18 modules. We first did miRNA enrichment analysis for both the modules enriched by the miRNA clusters, and the miRNA clusters enriched by the modules. 14 of 46 modules identified with our method were enriched (Bonferroni corrected *p*-value <0.05) and 8 clusters were enriched by the modules by setting the overlap of the clusters and modules being 3 or larger. In contrast, there is one module enriched by miRNA clusters, and one cluster enriched by modules for the modules identified by Mirsynergy. We also did Gene Ontology biological process (GO-BP) terms and KEGG pathway enrichment analysis. 15 modules are enriched by GO-BPs and 7 modules are enriched by KEGG pathways with our method, while 4 modules are enriched by GO-BPs and one is enriched by KEGG pathway. This may be because the interactions of genes and miRNAs are very sparse in our data set, which results in similar synergy scores of many genes/miRNAs and thus one module may consist of many genes/miRNAs, while other modules have very small size. As shown in Table 4, although the average number of genes and the average number of miRNAs of the modules identified by Mirsynergy are larger than that of our method, there is one module having 1152 genes. Such cases have been addressed in [43]. By taking into account the coexpressions of miRNAs, the miRNAs that may compose modules are more densely connected, which can be identified with our method with a high accuracy. The identified modules by Mirsynergy are in Additional file 2, and the enrichment results are in Additional file 4.

## Discussion

MiRNAs are actively involved in many biological processes by regulating the post-transcriptional gene expression. Increasing evidence shows that miRNAs play critical roles in many diseases including cancer and have a potential clinical value in diagnosis, treatment and prognosis. Although many works have been done to identify the targets of miRNAs and elucidate their complex regulatory networks, the complex relationships between miRNAs and genes are not fully understood. In this paper, we integrated the gene expression and miRNA expression data to study their complex interactions. By computing the pairwise Pearson correlation coefficients, we transformed the two data sets into networks. Then we proposed an optimization model to identify the modules in the integrated networks. We define the modules as subnetworks composed of genes, miRNAs, gene-gene interactions, miRNA- miRNA interactions, and gene-miRNA interactions. With such definitions, we found the interaction patterns of genes and miRNAs in the complex network. An approximate numerical algorithm is developed to solve the optimization problem. Compared to the existing methods, our method considers both the interactions within gene-gene, miRNA-miRNA networks, and the interactions between gene and miRNAs. By tuning the parameters for intra- and inter- networks, our method can give a good balance of all the interactions. The proposed method can be extended to study the modules in more networks with inter-connections. One weakness of our method is that the number of modules *K* should be given. To find the consistent results, we should try different *K*Šs, which may waste some computational time. Also, in other real applications, the identification accuracy may be related to the density of the intra-network connections. Thus we may need to add more tuning parameters to balance the intra-network connections. We applied our proposed method to an ovarian cancer data set. 14 modules are enriched by the miRNA clusters with overlap size being at least 3, 15 modules are enriched by GO-BP terms, and 7 modules are enriched by KEGG pathways significantly. In the identified modules, 122 miRNAs are cancer associated and 29 miRNAs are related to ovarian cancer, which has a *p*-value 0.039. These results show that the genes and miRNAs act together to contribute to the cancers. To find the omarkers of cancers, or develop therapy methods for cancers, we should take into account their interactions. From the module structures, we can also predict the unknown gene-miRNA interactions based on the known gene-miRNA interactions. These predicted results may give some theoretical basis for further experimental validations. Although with our current method we can get more information on gene-miRNA interactions, their complex relationships are far from being fully known. To understand the biological system better, we need to add more elements into the model. Integrating with other data sets, such as DNA methylation, histone modification, is left as one of our research topics.

## Conclusions

Our proposed method provides a way for studying the module structures in the complex gene-miRNA interaction network. The experimental results show that the modules composed of both genes, miRNAs, and their interactions are very likely to be related to cancers. These identified modules provide important information for further cancer studies, and are worth experimental validations.

## Additional files

Additional file 1 The identified modules. We listed all the 46 modules identified with our proposed method. Each module includes both genes and microRNAs. (CSV 51 kb)

Additional file 2 The identified modules with Mirsynergy. We listed all the modules identified with Mirsynergy. Each module includes both genes and microRNAs. (CSV 87 kb)

Additional file 3 Enrichment results of the modules in Additional file 1. We presented the GO-BP enrichment results and KEGG pathway enrichment results of modules in Additional file 1. (XLSX 120 kb)

Additional file 4 Enrichment results of the modules in Additional file 2. We presented the GO-BP enrichment results and KEGG pathway enrichment results of modules in Additional file 2. (XLSX 68 kb)
